# What Does Women’s Facial Attractiveness Signal? Implications for an Evolutionary Perspective on Appearance Enhancement

**DOI:** 10.1007/s10508-021-01955-4

**Published:** 2021-03-17

**Authors:** Benedict C. Jones, Alex L. Jones, Victor Shiramizu, Claire Anderson

**Affiliations:** 1 School of Psychological Sciences and Health, University of Strathclyde, Glasgow, G4 0LN Scotland; 2 Department of Psychology, Swansea University, Swansea, Wales; 3 Institute of Psychology & Neuroscience, University of Glasgow, Glasgow, Scotland; 4 Textile Design Programme, Chelsea College of Arts, University of the Arts London, London, England

In their Target Article, Davis and Arnocky (2020) suggest that evolutionary theories of mate preferences can contribute to our understanding of why appearance-enhancement behaviors are seemingly ubiquitous. We support their argument that an interdisciplinary approach, in which evolutionary and other perspectives are fully integrated, will give us a more complete understanding of appearance-enhancement behaviors. We also agree that evolutionary theories of mate preferences have the potential to provide new insights into why such behaviors are so common. Here, we use the literature on women’s facial attractiveness to highlight an important limitation of this argument: uncertainty about precisely what is signalled by physical attractiveness.

Davis and Arnocky (2020) suggest that women employ behaviors such as the use of cosmetics to enhance attractive facial characteristics (e.g., Russell, 2010). They suggest women engage in such behaviors primarily to increase their attractiveness to potential mates and romantic partners. Davis and Arnocky also argue that, in doing so, women exaggerate facial characteristics that have been shaped by sexual selection to advertise aspects of their underlying physical condition (Batres et al., 2019; Jones & Kramer, 2015).

Davis and Arnocky’s (2020) argument complements and builds on influential evolutionary theories of facial attractiveness. However, it also raises the question of what specific aspects of women’s underlying physical condition are signalled by attractive facial characteristics. This issue bears directly on Davis and Arnocky’s proposal that evolutionary theories of mate preferences can offer new insight into *why* appearance-enhancement behaviors are so common (i.e., that these theories can offer new insight into *ultimate* explanations for such behaviors). With this point in mind, we discuss four aspects of underlying physical condition typically emphasized and considered by evolutionary theories of facial attractiveness.

Our discussion highlights the inconsistent results that are typical of work on putative correlations between facial attractiveness and women’s physical condition. Although we focus on women’s facial attractiveness in our Commentary, we note here that a similar problem (inconsistent results for associations between attractiveness and physical condition) is also evident in the literature on women’s body attractiveness (Bleske-Rechek et al., 2011; Grillot et al., 2014; Jasieńska et al., 2004; Jones et al., 2018) and in work on men’s physical attractiveness (reviewed in Jones et al., 2019).

## Susceptibility to Infectious Illnesses

Susceptibility to infectious illnesses is perhaps the underlying aspect of physical condition most commonly assumed to be signalled by women’s facial attractiveness (Little et al., 2011; Thornhill & Gangestad, 1999). While robust correlations between facial attractiveness and measures of susceptibility to infectious illnesses are arguably a very strong prediction of sexual selection theory, we agree with Davis and Arnocky’s (2020) stated view that evidence for such correlations in young adult women is equivocal.

Several studies of women’s facial attractiveness have reported that women with more attractive faces report fewer past health problems (Gray & Boothroyd, 2012; Hume & Montgomerie, 2001; Shackelford & Larsen, 1999) or that attractive facial characteristics, such as prototypicality, are related to self-reported health (Jones, 2018; Rhodes et al., 2001). However, these findings have not been replicated in other studies, many of which tested considerably larger samples than the studies described above (e.g., Cai et al., 2019; Kalick et al., 1998; Thornhill & Gangestad, 2006). Similarly, although one study (Rantala et al., 2013) reported that women with relatively unattractive faces had higher cortisol (a potential marker of immunosuppression), subsequent studies did not replicate this finding (Gonzalez-Santoyo et al., 2015; Han et al., 2016). Studies that assessed measures of immune function also found little compelling evidence that immunocompetence and women's facial attractiveness were significantly correlated (Cai et al., 2019; Foo et al., 2017).

While it would be premature to conclude susceptibility to infectious illnesses and facial attractiveness are not correlated, the literature does not appear to show the robust consistent correlations one would expect to see if susceptibility to illnesses underpinned (or contributed substantially) to women’s facial attractiveness. However, it is important to note here that, while many studies have tested for possible links between susceptibility to infectious illnesses and facial attractiveness, very few have directly investigated the effects of current infections on facial attractiveness. Studies that have investigated this issue have found individuals with infections display facial characteristics that are considered unattractive, such as paler lips and skin (Axelsson et al., 2018). Thus, it is possible that appearance-enhancement behaviors function to obscure facial cues of current illness (e.g., pallor and fatigue cues), rather than enhance signals of good long-term health.

## Sex Hormones

Another aspect of women’s physical condition that is often emphasized in evolutionary theories of facial attractiveness is levels of estrogen and progesterone (Little et al., 2011; Thornhill & Gangestad, 1999). However, and as discussed above for susceptibility to infectious illnesses, empirical evidence for the proposal that women with more attractive faces tend to have higher levels of these hormones is mixed.

Law Smith et al. (2006) reported a significant positive correlation between ratings of women’s facial attractiveness and estradiol. They also reported a positive correlation between facial attractiveness and progesterone, although this relationship was not significant. By contrast with these results, neither Puts et al. (2013) nor Jones et al. (2018) found that women with more attractive faces had higher levels of estradiol or progesterone. Furthermore, Law Smith et al. reported that the use of cosmetics might actually obscure links between attractiveness and hormone levels. If robust, this finding would be difficult to reconcile with the idea that appearance-enhancement behaviors increase the visibility of hormone-linked facial characteristics.

Given the null results from two recent studies with large same sizes, it seems unlikely that adult levels of sex hormones are an important factor in women’s facial attractiveness. Thus, it is also unlikely that appearance-enhancement behaviors specifically target hormone-linked facial characteristics. Of course, such null results do not speak to the question of whether facial attractiveness in adulthood is related to hormone levels experienced during puberty or in utero. Such studies may be an important direction for future research into possible hormonal influences on attractive facial characteristics.

## Position in the Menstrual Cycle

Many researchers have suggested that women’s facial attractiveness increases during the fertile (i.e., late follicular) phase of the menstrual cycle. However, although such results are widely cited as strong evidence that sexual selection has shaped women’s facial attractiveness, evidence for this phenomenon is mixed.

Roberts et al. (2004) found that face photographs of women taken during the follicular (high-fertility) phase of the menstrual cycle were rated as more attractive than face photographs of women taken during the luteal (low-fertility) phase of the menstrual cycle. However, in a study using a nearly identical methodology, Bleske-Rechek et al. (2011) did not replicate this effect of fertility on facial attractiveness. Findings from studies investigating whether women’s facial attractiveness tracks changes in hormone levels have presented more consistent evidence that women’s facial attractiveness is influenced by cyclic changes in their fertility (Jones et al., 2018; Puts et al., 2013). It is important to note, however, that the extent of the changes in attractiveness is extremely small in those studies that do report significant changes in facial attractiveness during the menstrual cycle. Consequently, it is unlikely that cyclic changes in facial attractiveness would have the type of important effect on women’s facial attractiveness relative to other women that would be necessary to increase their attractiveness to potential mates (see Havlicek et al., 2015 for detailed discussion of this issue). Thus, it seems unlikely that appearance-enhancement behaviors target facial characteristics linked to cyclic changes in fertility, particularly given appearance-enhancement behaviors do not reliably track changes in fertility during the menstrual cycle (Arslan et al., 2018). Studies investigating cyclic changes in women’s facial coloration (Burriss et al., 2015; Jones et al., 2015) and shape information (Marcinkowska & Holzleitner, 2020; Oberzaucher et al., 2013) have also reported somewhat mixed results.

The studies described above typically tested for changes in women’s attractiveness when women were not using cosmetics and when non-facial cues (e.g., hairstyle, clothing) were not visible. Although some researchers have suggested that women might be more likely to engage in appearance-enhancing behaviors during the fertile phase of the menstrual cycle (Haselton & Gildersleeve, 2011), these findings have not been replicated in subsequent work with larger samples (Arslan et al., 2018).

## Youth

As Davis and Arnocky (2020) note, men’s preferences for younger adult women are evident across diverse societies. Because women with attractive faces are perceived to be youthful, it is plausible that appearance-enhancement behaviors exaggerate attractive facial characteristics because they signal youth. However, this does not necessarily indicate why youthful-looking facial characteristics are perceived to be attractive in women. The dominant theory regarding men’s preferences for youthful facial characteristics in women is that youthful women have greater reproductive potential (i.e., are likely to be able to produce more children). Although there have been few direct tests of this claim, Bovet et al. (2018) found that women’s facial attractiveness was positively correlated with their expected age of menopause (assessed from maternal age at menopause). Replications and extensions of this type of study might be a fruitful avenue for research examining underlying aspects of physical condition signalled by women’s facial attractiveness. There is also some evidence that appearance-enhancement behaviors do not alter perceptions of youth, per se. For example, Russell et al. (2019) found that, although women between 40 and 50 years of age were judged to be younger when wearing makeup than when no wearing makeup, women around 20 years of age were judged to be older when wearing makeup than when no wearing makeup. Indeed, makeup use appears to explain only 2% of the variability in women’s facial attractiveness (Jones & Kramer, 2015).

## Conclusion

In summary, despite the popularity of the argument that women’s facial attractiveness signals aspects of physical condition that are important in the context of mate choice, evidence for this claim is rather mixed and results are far from clear. Thus, while it may well be the case that behaviors that enhance women’s facial appearance do so by exaggerating attractive facial characteristics, we suggest that there is actually relatively little compelling evidence that they specifically target facial characteristics that signal underlying aspects of physical condition. Furthermore, the specific aspects of physical condition that attractive physical characteristics in women’s faces actually signal remain mysterious. We suggest that more rigorous work investigating how the aspects of women’s facial attractiveness that are exaggerated by appearance-enhancement behaviors relate to underlying aspects of physical condition is needed. Such work would clarify the contribution evolutionary theories of facial attractiveness can make to our understanding of why such appearance-enhancement behaviors appear to be ubiquitous.

## Understanding Appearance Enhancement in the Digital Domain

Models of appearance enhancement have typically focused on explaining the motivation behind appearance-enhancement behaviors as they apply to face-to-face, real-world interactions. However, with increasing amounts of social interaction taking place online, models of appearance enhancement will also need to be able to account for the range of appearance-enhancement behaviors that are applied to digital, online interactions. For example, L'Oréal Paris cosmetics recently launched the virtual makeup range, “Signature Faces,” which acts as hyper-real filters for Zoom, Microsoft Teams, Google Hangouts, and social media apps (Buller & Panesar, 2020). Indeed, there has recently been an explosion of interest in such methods from both users and product designers, as people experiment with the boundaries of digital aesthetics and cosmetics (Fairs, 2020). Whether the motivations behind the application of digital methods for appearance enhancement are broadly the same or qualitatively different to those implicated in real-world interactions is an exciting avenue for research in this area. Indeed, as the forum for appearance-enhancement behaviors moves further away from the specific type of interactions that are likely to have been shaped by sexual selection, it is possible that evolutionary theories of mate preferences could become increasingly irrelevant to our understanding of appearance enhancement.
